# Timp1 Promotes Cell Survival by Activating the PDK1 Signaling Pathway in Melanoma

**DOI:** 10.3390/cancers9040037

**Published:** 2017-04-21

**Authors:** Mariana Toricelli, Fabiana H. M. Melo, Aline Hunger, Daniela Zanatta, Bryan E. Strauss, Miriam G. Jasiulionis

**Affiliations:** 1 Pharmacology Department, Universidade Federal de São Paulo, São Paulo 04039-032, Brazil; maritoricelli@gmail.com (M.T.); fabimmelo@yahoo.com.br (F.H.M.M.); 2 Center for Translational Investigation in Oncology/LIM 24, Cancer Institute of São Paulo, School of Medicine, University of São Paulo, São Paulo 01246-000, Brazil; linehunger@gmail.com (A.H.); danizanatta@gmail.com (D.Z.); bstrauss@usp.br (B.E.S.)

**Keywords:** Timp1, *anoikis* resistance, PI3K pathway, PDK1, PKC, melanoma

## Abstract

High TIMP1 expression is associated with poor prognosis in melanoma, where it can bind to CD63 and β1 integrin, inducing PI3-kinase pathway and cell survival. Phosphatidylinositol (3,4,5)-trisphosphate (PIP3), generated under phosphatidylinositol-3-kinase (PI3K) activation, enables the recruitment and activation of protein kinase B (PKB/AKT) and phosphoinositide-dependent kinase 1 (PDK1) at the membrane, resulting in the phosphorylation of a host of other proteins. Using a melanoma progression model, we evaluated the impact of Timp1 and AKT silencing, as well as PI3K, PDK1, and protein kinase C (PKC) inhibitors on aggressiveness characteristics. Timp1 downregulation resulted in decreased *anoikis* resistance, clonogenicity, dacarbazine resistance, and in vivo tumor growth and lung colonization. In metastatic cells, pAKT^Thr308^ is highly expressed, contributing to *anoikis* resistance. We showed that PDK1^Ser241^ and PKCβII^Ser660^ are activated by Timp1 in different stages of melanoma progression, contributing to colony formation and *anoikis* resistance. Moreover, simultaneous inhibition of Timp1 and AKT in metastatic cells resulted in more effective *anoikis* inhibition. Our findings demonstrate that Timp1 promotes cell survival with the participation of PDK1 and PKC in melanoma. In addition, Timp1 and AKT act synergistically to confer *anoikis* resistance in advanced tumor stages. This study brings new insights about the mechanisms by which Timp1 promotes cell survival in melanoma, and points to novel perspectives for therapeutic approaches.

## 1. Introduction

Cutaneous melanoma is a melanocytic tumor whose incidence and mortality are on the rise worldwide. Although melanoma accounts for only 4% of all skin cancers, its mortality rate corresponds to 74% of those related to skin cancer in general [1]. The overall incidence is 160,000 cases, with 48,000 deaths per year [1].

MAPK signaling pathway (mitogen activated protein kinase), including the cascade of proteins NRAS, BRAF, MEK1/2, and ERK1/2, is one of most-studied signaling pathways in melanoma. This path is considered the main route changed in melanoma and is involved in cell survival, proliferation, and migration, and is related to both development and melanoma progression [2]. So far, the best characterized effectors of RAS proteins are RAF kinase and phosphatidylinositol-3-kinase (PI3K) [3].

The phosphatidylinositol-3-kinase (PI3K) pathway is one of the most important pathways in cancer metabolism and growth. One of the key effector proteins in this pathway is known as PKB (protein kinase B) or AKT, which has three isoforms (AKT1, AKT2, and AKT3), with AKT3 being found frequently activated in melanomas [3]. By phosphorylation of several intracellular substrates, AKT can modulate various biological processes, such as survival, cell cycle progression, growth, and cell metabolism. AKT activation is initiated when the translocated AKT is phosphorylated at threonine 308 (Thr308) by phosphoinositide-dependent kinase 1 (PDK1) [4]. However, the complete activation occurs only when serine 473 is phosphorylated by the mTORC2 complex [5]. Phosphoinositide-dependent kinase 1 (PDK1) is a proximal signaling molecule of phosphatidylinositol-3-kinase, which is required for metabolic activation [6]. Many other kinases, in addition to AKT, are known to be targets of PDK1 and have attracted great interest in the scientific community. Among them are glucocorticoid-dependent kinase (SGK), p70 ribosomal S6 protein kinase (S6K), p90 ribosomal protein S6 kinase (RSK), and atypical PKC isoforms [6]. Du and coworkers revealed that mammary-specific ablation of PDK1 could delay tumor initiation, progression, and metastasis in a spontaneous mouse tumor model [7]. They also demonstrated that inducible deletion of PDK1 could noticeably shrink the growing breast tumors. All of these results were obtained without AKT involvement [7].

In our laboratory, we developed a model to study different stages of melanoma progression. Murine melanocytes were subjected to sequential cycles of substrate adhesion impediment, thereby obtaining different cell lines representing pre-malignant melanocytes, non-metastatic melanoma, and metastatic melanoma [8]. These cell lines show differences in the expression of a variety of genes and proteins, including Timp1 [9]. Timp1 is a member of the family of matrix metalloproteinase inhibitors, which contains four members (Timp1, Timp2, Timp3, and Timp4) [10]. Tissue inhibitors of metalloproteinases (TIMPs) are multifaceted molecules that exhibit properties beyond their classical proteinase inhibitory function. In our model, we demonstrated a progressive increase in Timp1 expression along the melanoma progression [9]. It was demonstrated that Timp1 confers higher survival, since melanocytes overexpressing Timp1 become able to resist *anoikis* and form colonies in soft agar [9]. Moreover, melanoma cells overexpressing Timp1 acquire increased capacity to metastasize in vivo [9]. In addition, we reported, for the first time, the assembly of a supramolecular complex containing Timp1, CD63, and β1-integrins at the cell surface in melanoma cells, and its involvement in the acquisition of an *anoikis*-resistant phenotype through the PI3K signaling pathway [11]. However, the signaling pathway induced by Timp1 to protect melanoma cells from apoptosis is still unknown. In the present study, we have investigated the mechanisms underlying Timp1 role in melanoma progression. We present evidence that Timp1 overexpression increases PDK1 phosphorylation at serine 241 and PKCβII at serine 660, and this increase results in an *anoikis* resistance phenotype. We also observed that the simultaneous deletion of Timp1 and AKT prevents more efficiently cell survival, providing a possible new therapeutic strategy for metastatic melanoma.

## 2. Results

### 2.1. Timp1 Silencing Results in Decreased Cell Survival in Vitro along with Melanoma Progression

**Figure 1:**
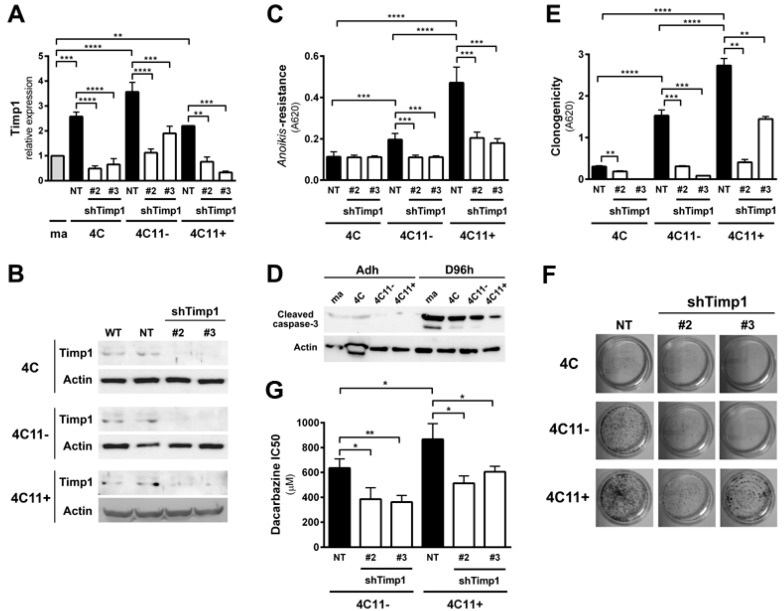
Downregulation of Timp1 decreases cell survival in melanoma cells. (**A**,**B**) Timp1 mRNA expression was assessed by RT-qPCR and Western blotting, respectively, in different cell lines representing different stages of melanoma progression after transduction with viral particles containing two different shRNA sequences for Timp1 (shTimp1#2 and shTimp1#3) or control non-target shRNA (non-target, NT); (**C**,**D**) cell lines were maintained in suspension for 96 h and the number of viable cells was estimated, respectively, using MTT and analysis of cleaved caspase-3 by Western blotting; (**E**,**F**) 200 cells were incubated at 37 °C on 60 mm^2^ plates for 10 days. After this period, the clone formation was, respectively, quantified and visualized after toluidine blue staining; and (**G**) the non-metastatic 4C11− and metastatic melanoma cell line 4C11+ were treated for 48 h with dacarbazine (IC50). After this period, cell viability was analyzed by MTT assay. ma: melan-a melanocytes; 4C: pre-malignant melanocytes; 4C11−: non-metastatic melanoma cell line; 4C11+: metastatic melanoma cell line; NT: control non-target shRNA; shTimp1#2: clone 2 silenced for Timp1; shTimp1#3: clone 3 silenced for Timp1. * *p* < 0.05, ** *p* < 0.01, *** *p* < 0.001, **** *p* < 0.0001.

Previous data from our laboratory showed increased Timp1 expression along melanoma progression and more important the assembly of a supramolecular complex containing Timp1, CD63, and β1-integrins associated with a more aggressive phenotype [9,11]. To further analyze the role of Timp1 along the progression of melanoma, shRNA technology was used for silencing Timp1 in cell lines representing different stages of melanoma progression: pre-malignant melanocytes (4C), non-metastatic melanoma cells (4C11−), and metastatic melanoma cells (4C11+) (Figure 1A,B). As previously demonstrated, Timp1 overexpression confers an *anoikis*-resistant phenotype to both the murine melan-a melanocyte lineage and melanoma cell lines [9,11]. To further elucidate the performance of Timp1 in providing greater capacity for survival, we conducted the analysis of *anoikis* resistance in 4C, 4C11−, and 4C11+ cell lines silenced for Timp1. Only the melanoma cell lines 4C11− and 4C11+ were sensitized to cell death via *anoikis* in the absence of Timp1, as shown by MTT assay (Figure 1C) and caspase-3 cleavage (Figure 1D) after adhesion impediment. Regarding colony formation capacity (clonogenicity), a decrease was noted in all cell lines examined, strengthening the role of Timp1 in cell survival in vitro (Figure 1E,F). In addition, the relevance of Timp1 in apoptosis induced by others factors was evaluated by treating melanoma cells with dacarbazine, a chemotherapeutic drug used in metastatic melanomas [12]. The half-maximal inhibitory concentration (IC50) of dacarbazine was determined in melanoma cells silenced for Timp1. In our model, increased sensitivity to dacarbazine was observed in melanoma cells silenced for Timp1 (Figure 1G).

### 2.2. Timp1 Silencing Reverses the Aggressive Phenotype of Metastatic Melanoma Cells In Vivo

**Figure 2:**
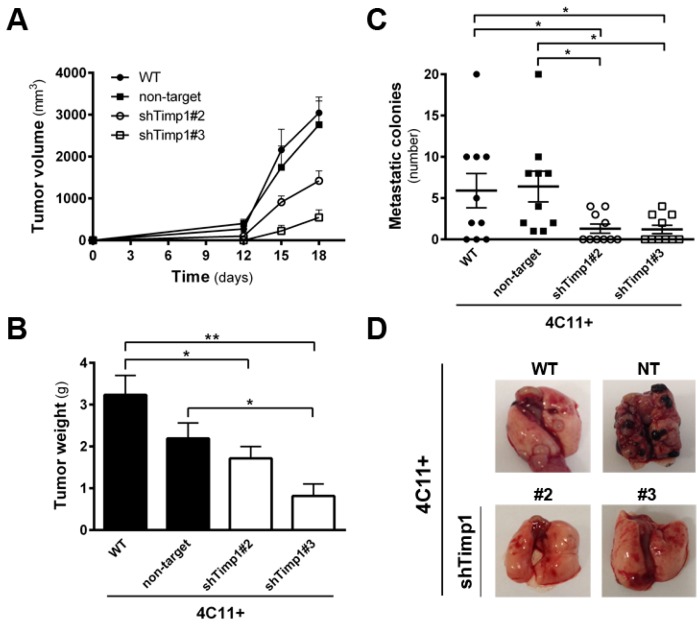
Timp1 down-expression decreases in vivo tumor growth and lung colonization. Metastatic melanoma cells 4C11+ (2 × 10^5^) were injected subcutaneously into C57BL/6 mice (groups of five animals) and the tumor volume (**A**) and weight (**B**) were determined; and (**C**,**D**) for experimental metastasis assay, 2.5 × 10^5^ cells were injected in the lateral tail vein of C57BL/6 mice. Mice were sacrificed 21 days after injection, their lungs were removed, and the number of metastatic colonies in the lung was determined. WT: wild-type 4C11+ metastatic melanoma cell line; non-target (NT): control non-target shRNA; shTimp1#2: clone 2 silenced for Timp1; shTimp1#3: clone 3 silenced for Timp1. Representative graphics of two independent experiments yielding similar results are shown. * *p* < 0.05, ** *p* < 0.01.

Since the silencing of Timp1 conferred sensitivity to *anoikis* and reduced the colony formation along melanoma progression, we analyzed whether reducing Timp1 levels would affect tumor growth and lung colony formation in vivo. For this purpose, we injected metastatic melanoma 4C11+ cell line, wild-type and silenced for Timp1, in the subcutaneous tissue of female C57BL/6 mice and observed tumor growth. The 4C11+ cell line is extremely aggressive and, after 12 days, we noted tumor growth in animals inoculated with control cells, but not with Timp1 silenced cells. Despite the expected variability found in in vivo experiments, animals inoculated with both Timp1 silenced clones presented a lag time of 14 days for the appearance of tumor mass (Figure 2A). On day 18, tumor weight was measured and animals were sacrificed (Figure 2B). We observed a significant reduction in both the volume and weight of tumors from animals inoculated with the cell lines silenced for Timp1.

Our laboratory has shown that the increase in Timp1 expression provides greater efficiency in metastases development, indicating a correlation between levels of Timp1 and a worse prognosis for melanoma [9,11]. Moreover, various studies have correlated Timp1 increased expression in malignant progression and poor prognosis, both in humans and in experimental tumor models. We observed the effect of silencing Timp1 in the development of lung colonies after injecting 4C11+ intravenously. Cells were inoculated via the lateral tail of female C57BL/6 mice and, after 21 days, the lungs were analyzed for the presence of colonies (Figure 2C,D). We observed that animals inoculated with silenced Timp1 clones exhibited less lung metastatic nodules.

### 2.3. AKT Contributes to Anoikis Resistance in Metastatic Melanoma Cell Line Independently of Timp1

**Figure 3:**
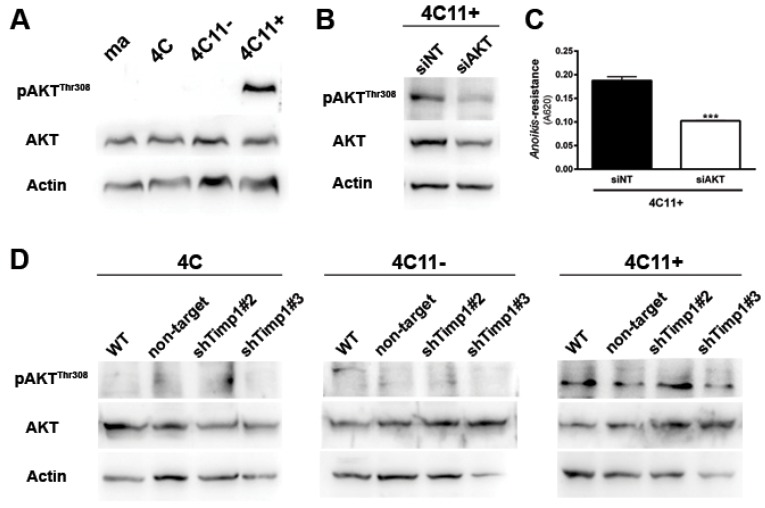
AKT contributes to *anoikis* resistance in metastatic melanoma cell lines independently of Timp1. (**A**) AKT activation was evaluated in melan-a (ma), 4C, 4C11− and 4C11+ cells lines by Western blotting. As loading control, total AKT and β-actin were used. (**B**) AKT activation was analyzed by Western blotting in the 4C11+ cell line, treated with a non-target siRNA (siNT) and a siRNA specific to AKT (siAKT). Total AKT and β-actin were used as loading controls. (**C**) *Anoikis* resistance was determined after maintaining control (siNT) and siAKT 4C11+ cells in suspension for 96 h. (**D**) AKT activation was analyzed by Western blotting in 4C, 4C11−, and 4C11+ cell lines and their Timp1 silenced clones. Total AKT and β-actin (Actin) were used as loading controls. WT: non-treated cells; non-target: control non-target shRNA; shTimp1#2: clone 2 silenced for Timp1; shTimp1#3: clone 3 silenced for Timp1. *** *p* < 0.001.

Since AKT is a major downstream effector of PI3K in various cell types [2] and an important regulator of cell survival, we analyzed AKT activation along the melanoma progression. For this, we verified the activation pattern of AKT at threonine 308 (AKT^Thr308^) by Western blot assay. We can see that only metastatic melanoma cell line 4C11+ has activated AKT (Figure 3A). To demonstrate the real impact of AKT in the metastatic 4C11+ cell line, we inhibited AKT through RNA interference technology (Figure 3B) and checked if any change would occur in the *anoikis* sensitivity. In Figure 3C, we found that the reduction of AKT rendered cells less able to resist to *anoikis*. As AKT is activated only in the metastatic melanoma 4C11+ cell line (Figure 3A) and this activation was shown to contribute to *anoikis* resistance (Figure 3C), we checked whether there would be a correlation between AKT and Timp1. For this, we analyzed AKT activation in the Timp1 silenced cell lines. As shown in Figure 3D, there is no change in AKT activation in melanoma cell lines silenced for Timp1.

### 2.4. PDK1 Is Activated by Timp1 along Melanoma Progression and Contributes to Cell Survival

**Figure 4:**
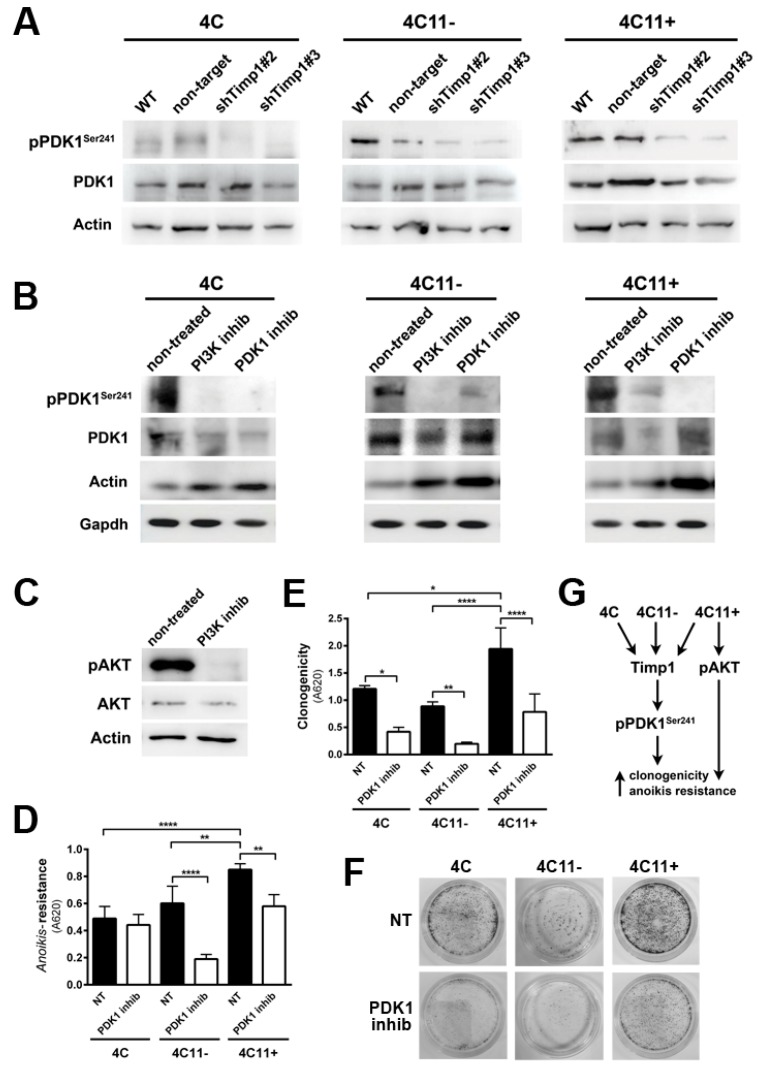
PDK1 is activated by Timp1, along with melanoma progression, and contributes to cell survival. (**A**) PDK1 activation was examined in cell lines 4C, 4C11−, and 4C11+ and their Timp1-silenced clones. Total PDK1 and β-actin were used as loading controls. WT: non-treated cells; non-target: control non-target shRNA; shTimp1#2: clone 2 silenced for Timp1; shTimp1#3: clone 3 silenced for Timp1; (**B**) cell lines treated with LY294002 (PI3K inhibitor), GSK2334470 (PDK1 inhibitor), and Bisindolylmaleimide I (PKC inhibitor) were analyzed for PDK1 activation by Western blotting. Total PDK1, β-actin (Actin), and GAPDH were used as loading controls; (**C**) 4C11+ metastatic melanoma cells treated or not with LY294002 (PI3K inhibitor) were analyzed for AKT activation by Western blotting. β-actin was used as a loading control; (**D**) cell lines non-treated (NT) or treated with GSK2334470 (PDK1 inhibitor) were evaluated for *anoikis* resistance after maintained in suspension for 96 h; (**E**,**F**) colony formation capacity was determined in cell lines non-treated (NT) or treated with GSK2334470 (PDK1 inhibitor); and (**G**) cell lines corresponding to both early (4C and 4C11−) and late (4C11+) stages of melanoma progression confer cell survival via Timp1 by activating PDK1 pathway. In metastatic cell line (4C11+), AKT also contributes to *anoikis* resistance. * *p* < 0.05, ** *p* < 0.01, *** *p* < 0.001.

We found that Timp1 is highly expressed from the pre-malignant 4C cell line to metastatic cell line 4C11+ and that this high expression leads to increased *anoikis* resistance and clonogenic capacity. We also observed that higher levels of Timp1 do not alter the AKT activation in metastatic cells, thus showing no correlation between the increase in Timp1 and AKT phosphorylation. To elucidate which molecule(s) in the PI3K pathway is modulated by Timp1, we analyzed PDK1, a molecule activated after being recruited to PIP3, in the cell lines silenced for Timp1. Recently, several studies have investigated the role of PDK1 in tumors and there is evidence that PDK1 can activate other molecules involved in cell survival, senescence, and proliferation independent of its AKT phosphorylating activity [13,14,15]. We observed a decreased PDK1 activation in pre-malignant 4C cell line and in melanoma cell lines 4C11− and 4C11+ silenced for Timp1 (Figure 4A).

One of the main roles of Timp1 in our melanoma progression model is to confer *anoikis* resistance. The above data indicate that Timp1 is related to the PDK1 activation, which could be related to *anoikis* resistance. To confirm this hypothesis, we submitted cell lines to the treatment with a PDK1 inhibitor, GSK2334470 (Figure 4B), and evaluated the *anoikis* survival rate (Figure 4D) and the ability to form colonies (Figure 4E,F). Figure 4B also confirms that PDK1 is a downstream target of PI3K in this model. The efficiency of the PI3K inhibitor, LY294002, in abrogating AKT activation is shown in Figure 4C. These data show that Timp1 confers cell survival by activating PDK1 along melanoma progression and that in metastatic cells AKT activation also contributes to *anoikis* resistance (Figure 4G).

### 2.5. Timp1 Modulates the PKC Activation via PDK1 in the Early Stages of Melanoma

**Figure 5:**
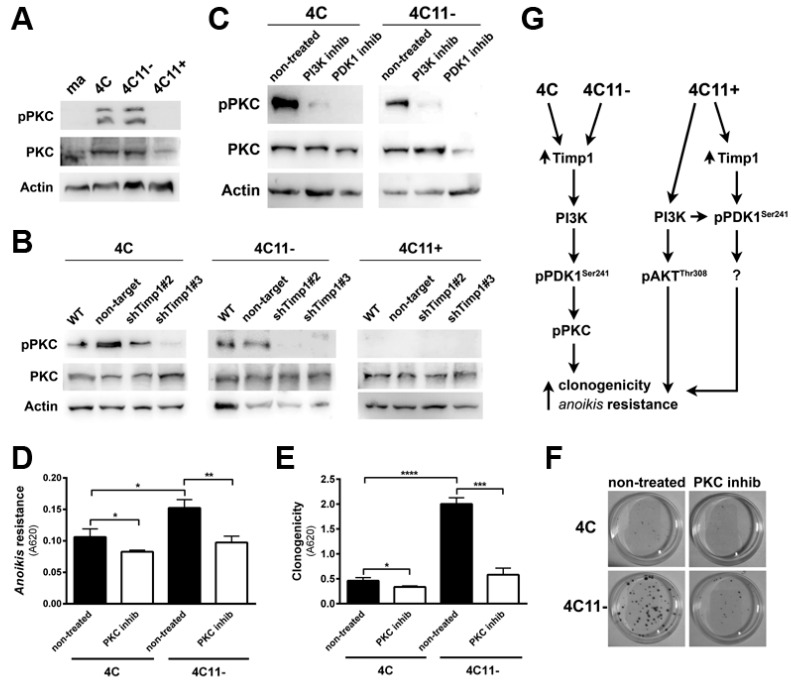
Timp1 modulates the PKC activation via PDK1 in the early stages of melanoma. (**A**) PKC activation was analyzed by Western blotting in the melan-a (ma), 4C, 4C11−, and 4C11+ cell lines. Total PKC and β-actin (Actin) were used as loading controls. (**B**) PKC activation was analyzed by Western blotting in cell lines silenced for Timp1. Total PKC and β-actin (Actin) were used as loading controls. WT: non-treated cell lines; non-target: control non-target shRNA; shTimp1#2: clone 2 silenced for Timp1; and shTimp1#3: clone 3 silenced for Timp1. (**C**) PKC activation was determined in cell lines (4C and 4C11−) treated with LY294002 (PI3K inhibitor) or GSK2334470 (PDK1 inhibitor). The control was subjected to the same conditions without any treatment (non-treated). Total PKC and β-actin (Actin) were used as loading controls. (**D**) *Anoikis* resistance was analyzed in pre-malignant 4C melanocytes and non-metastatic melanoma 4C11- cell lines, non-treated and treated with Bisindolylmaleimide I (PKC inhibitor). (**E**,**F**) Colony formation was evaluated in 4C and 4C11- cell lines, non-treated and treated with Bisindolylmaleimide I (PKC inhibitor). (**G**) Cell survival is favored by Timp1 in early stages of melanoma progression by activation of the PDK1/PKC pathway. In late stages (metastasis), both PDK1 activated by Timp1 and AKT independently of Timp1 contribute to cell survival. melan-a: melanocytes; 4C: pre-malignant melanocytes; 4C11−: non-metastatic melanoma cell line; 4C11+: metastatic melanoma cell line; * *p* < 0.05, ** *p* < 0.01, *** *p* < 0.001, **** *p* < 0.0001.

PDK1 is known to activate AKT at threonine 308, but it can also activate other molecules, such as PKC and SGK [15]. In this way, we analyzed PKC activation along melanoma progression. Interestingly, PKC was found to be activated in the early stages of melanoma, but not in metastatic cells (Figure 5A). To confirm that PKC is a downstream molecule of Timp1, we analyzed PKC activation in the cell lines silenced for Timp1. The cell lines presenting activated PKC (pre-malignant melanocytes 4C and non-metastatic melanoma cells 4C11−), but not metastatic cells, had reduced PKC activation when Timp1 was silenced (Figure 5B).

To confirm that PKC is a downstream target of PI3K and PDK1, we treated cell lines with specific inhibitors for PI3K and PDK1 (Figure 5C). As it was shown that the PKC is activated only in cell lines 4C and 4C11−, and that this activation is reversed in cell lines silenced for Timp1, the next step was to define the PKC role in cell survival. The cell lines were treated with a specific inhibitor of PKC, Bisindolylmaleimide I, and the number of cells surviving to *anoikis* and the ability to form colonies were analyzed. Pharmacological inhibition of PKC was able to reduce both the *anoikis* resistance (Figure 5D) and colony formation (Figure 5E,F). These data indicate that the role of the PI3K/PDK1/PKC pathway in cell survival induced by Timp1 in the early stages of melanoma progression, whereas in metastatic cells, both PDK1 induced by Timp1 and AKT, contribute to cell survival (Figure 5G).

### 2.6. Simultaneous Inhibition of Timp1 and AKT Impairs More Efficiently the Survival of Metastatic Melanoma Cells

**Figure 6:**
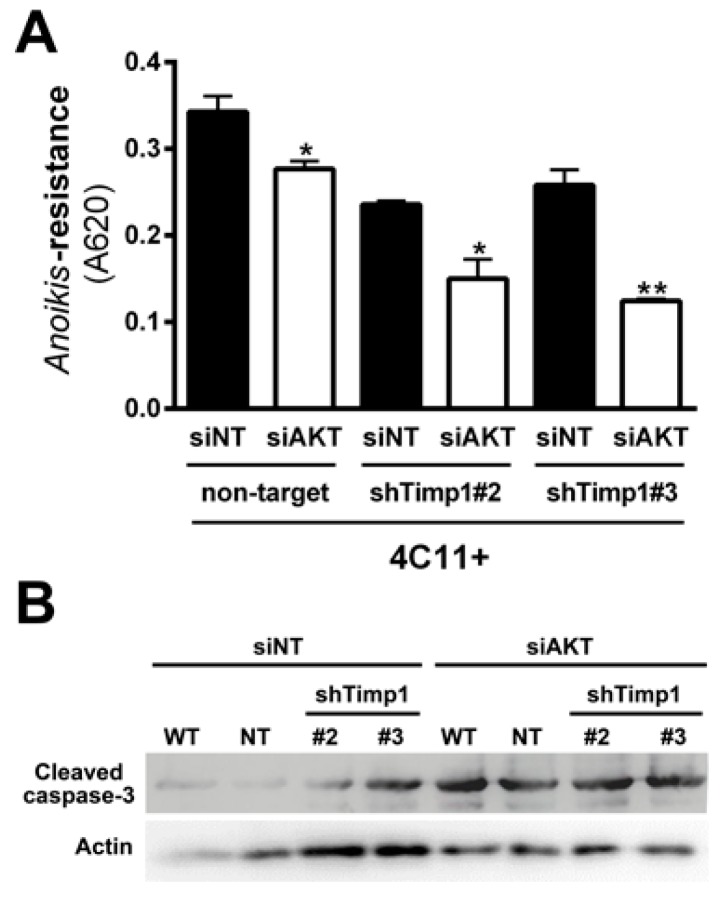
Simultaneous inhibition of Timp1 and AKT impairs more efficiently the survival of metastatic melanoma cells. The metastatic 4C11+ cell line silenced for Timp1 were silenced for AKT and analyzed for their capacity to survive (**A**) and induce caspase-3 cleavage (**B**); WT: non-treated cells; non-target (NT): control non-target shRNA; shTimp1#2: clone 2 silenced for Timp1; shTimp1#3: clone 3 silenced for Timp1. siNT: non-target siRNA; siAKT: siRNA specific for AKT. β-actin (Actin) was used as loading control. * *p* < 0.05, ** *p* < 0.01.

Our results demonstrated that decreasing Timp1 results in a less efficient *anoikis* resistance, colony formation and resistance to the drug dacarbazine in vitro (Figure 1). In vivo, Timp1 reduction was able to reduce dramatically tumor formation and the number of metastatic lung colonies (Figure 2). We also showed that Timp1 modulates phosphorylation of PDK1 and PKC along melanoma progression (Figure 4A and Figure 5B). In addition, AKT appeared to play a key role in the *anoikis* resistance in metastatic melanoma cells 4C11+ cells (Figure 3C). The next step was to determine whether the simultaneous inhibition of AKT and Timp1 would have a stronger effect on *anoikis* resistance in metastatic cells, since phosphorylated AKT is only found in these cells. For this purpose, we silenced AKT in 4C11+ metastatic cell line previously silenced for Timp1 and evaluated the *anoikis* resistance and caspase-3 cleavage. The simultaneous inhibition of Timp1 and AKT resulted in *anoikis* inhibition (Figure 6A) and increased caspase-3 cleavage (Figure 6B) in a more effective way.

## 3. Discussion

Previous work using a melanocyte malignant transformation model established by our group showed that increased expression of Timp1 along melanoma progression is correlated with *anoikis* resistance and malignant potential [9,11]. We also demonstrated that Timp1 associates differentially with CD63 and β1-integrins along melanoma progression and that this association may be related to the induction of the PI3K signaling pathway [11]. Moreover, we have shown that, in human metastatic melanoma cells, Timp1 and CD63 expression are increased and positively correlated with colony formation capability [11], strengthening the possible role of Timp1 in human melanoma genesis. Here we provide evidence for the mechanisms by which Timp1 contributes to melanoma progression through increasing cell survival.

We determined signaling pathways activated by Timp1 in cell lines corresponding to different phases of melanoma progression, performing a genetic approach using transfection assays to inhibit Timp1 gene expression. Decreasing Timp1 expression in 4C11− and 4C11+ melanoma cells resulted in less effective *anoikis* resistance, colony formation, chemotherapy resistance, in vivo tumor growth, and metastatic colony formation in the lung (Figure 1 and Figure 2), showing that Timp1 confers advantages which are essential for melanoma development, as it was shown for some other cancers. In epithelial breast cancer cells, TIMP1 induced TWIST1 expression, leading to E-cadherin downregulation and epithelial mesenchymal transition in a CD63-dependent manner [16]. A recent study showed that in hepatocellular carcinoma cells TIMP1 expression is associated with advanced TNM stage (classification of malignant tumors), intrahepatic metastasis, portal vein invasion, and vasculature invasion [17]. Bjerre et al. demonstrated that low expression of TIMP1 displayed significantly reduced sensitivity to the anti-estrogen fulvestrant and gene expression analysis revealed association between expression of TIMP1 and genes involved in metabolic pathways, epidermal growth factor receptor 1/cancer signaling pathways, and cell cycle [18]. In fact, different authors have confirmed a positive correlation of high TIMP1 expression with a poor prognosis in lung, brain, prostate, breast, colon, and several other cancers [19,20].

Aberrant PI3K/AKT signaling has been studied extensively in cancer and the relationship between the PI3K/AKT signaling pathway and tumor is clear. Dysregulation of the PI3K transduction pathway is associated with more than 50% of melanomas, contributing to malignant transformation and tumor progression by different mechanisms. We observed previously that Timp1 contributes to melanoma development activating PI3K signaling pathway since wortmaninn and LY inhibit *anoikis* resistance, a hallmark of cancer, acquired by melanocytes overexpressing Timp1 [11]. AKT, the main effector of the PI3K signaling pathway, was found to be activated in the 4C11+ metastatic melanoma cell line (Figure 3A) contributing to cell survival (Figure 3C). In human colon cancer cells, it was shown that TIMP1 positively regulates proliferation and renders tumor cells more resistant to apoptosis through the activation of FAK-PI3K/AKT and the MAPK signaling pathway [21]. Moreover, in human breast cancer cells, RNAi-mediated silencing of TIMP1 induced cell cycle arrest at the G1 phase associated with AKT and NFκB signaling pathway inhibition [20]. However, in our model, Timp1 is not involved in AKT activation since decreased Timp1 expression did not affect AKT phosphorylation in melanoma cells (Figure 3D). In the same way, AKT activation in melanocytes submitted to anchorage blockade was not affected by Timp1 overexpression [11]. Canonically, AKT is activated following growth and survival factors by a dual phosphorylation mechanism dependent on PI3K. However, it was recently shown that phosphoinositide 3-kinase-related kinases (PIKKs), including mammalian targets of rapamycin complex 2 (mTORC2), DNA-dependent protein kinase (DNA-PK), and ataxia telangiectasia mutated (ATM), are implicated in AKT regulation as a result of sustained stress conditions, including DNA damage and nutrient starvation [22,23,24]. Induction of DNA strand breaks recruits and activates DNA-PK to sites of DNA damage, which, in turn, stimulates AKT phosphorylation, promoting cell survival by induction of p21, a pro-survival transcription factor [23]. In fact, in our model p21 was shown to be phosphorylated by AKT and retained to the cytoplasm, where it plays a role in drug resistance [25]. The mechanisms associated with AKT activation independently of PI3K stimulation by TIMP1 in melanoma cells are under investigation.

It was also shown that the PI3K signaling pathway can act independent of AKT in tumorigenesis since PIK3CA mutant tumors have strikingly low levels of phosphorylated and activated AKT [26]. In human breast cancer cells, PI3K promotes anchorage-independent growth and colony formation in soft agar even in minimal AKT activation [27]. Activation of downstream targets via PI3K signaling is defined by substrate conformation and subcellular localization. A moderate amount of PDK1 at the membrane is sufficient to activate substrates in a PI3K-dependent manner, unlike AKT, which requires simultaneous protein-protein interactions following 3′-phosphatidylinositol synthesis. Following growth factor stimulation, PI3K is activated and PDK1 is found at the plasma membrane, where it binds to the phosphatidylinositol 3,4,5-trisphosphate [PtdIns(3,4,5)P3] second messenger which, in turn, can activate a group of kinases belonging to the AGC protein family, such as PKA, PKG, PKC, and GSK3 [28,29]. PDK1 expression, activation, and subsequent stimulation of downstream substrates can also be regulated by c-Jun transcription factor contributing to melanoma growth [30]. These enzymes regulate important processes of carcinogenesis, such as protein synthesis, metabolism, survival, and proliferation. PDK1 contributes to development of melanoma harboring the wild-type or knockout *Pten* genotype [14,31]. These authors also showed that inhibition of PI3K signaling effectively synergize with the PDK1 inhibitor, showing a novel therapeutic approach in melanoma treatment [14,31]. Interestingly, in our melanoma progression model, Timp1 was shown to modulate the PI3K/PDK1 axis, since decreased Timp1 expression and PI3K inhibition attenuated PKD1 phosphorylation along with melanoma genesis (Figure 4A). PDK1 contributes to cell survival since its pharmacological inhibition dramatically increased *anoikis* sensitivity and decreased colony formation (Figure 4D–F). These observations corroborate the data showing that PDK1 inhibitors might have therapeutic utility for cancer treatment. Recent studies also suggest that PDK1 inhibitors may have other benefits, including reduction of cancer cell resistance to chemotherapeutic drugs, such as Tamoxifen [32,33].

The PKC isoforms are activated by multiple mechanisms, including PI3K/PDK and MAPK kinases ERK and JNK after receptor tyrosine kinase and G protein coupled receptor stimulation in a variety of cells regulating innumerous biological process. Cell-specific functions performed by PKC enzymes are determined by a combination of different factors, including PKC isoform profiles, substrate specificity, subcellular localization, and protein interactions [34]. PKCα/β II and PCKδ, but not PKCξ, are phosphorylated in response to VEGF-mediated PI3K activation and contribute to vasculogenesis [35]. PKC activation is also associated with the regulation of both innate and adaptive immunity, being activated by Toll-like receptors, B-cell, and T-cell receptors [36]. In melanoma cells, PKC signaling pathway activation was found to be involved in both oncogenic or tumor suppressive effects [34]. Concomitant inhibition of PKC and p53-MDM2 or PKC and mTORC1 pathways efficiently decreased cell survival and increased apoptosis of uveal melanoma cells, providing a very attractive treatment [37]. However, overexpression of the PCKδ isoform in B16F10 melanoma cells resulted in suppressed ceramide production and induced cell death [38]. In our model we detected increased levels of phosphorylated PKC isoforms α, β, βII, δ, ε, η, and θ only in pre-malignant 4C cells and non-metastatic 4C11− cells (Figure 5A), which correspond to early stages of melanoma development. Activation of PKC is downstream a PI3K/PDK signal transduction, since PKC phosphorylation was abrogated in the presence of PI3K and PDK inhibitors (Figure 5C). It was shown that the PKC signaling pathway regulates TIMP1 expression in connective tissue following myocardium injury and inflammation, contributing to tissue remodeling [38]. In addition, increased PCK activity induced by phorbol-12-myristate-13-acetate (PMA) augments TIMP1 expression in colon cancer cells [39]. However, the regulation of PKC signal transduction by TIMP1 was not observed. Here, we demonstrated that PKC activation in early phases of melanoma genesis requires TIMP1 (Figure 5B) and it is critical to favor cell survival (Figure 5D–F). Activation of other signaling pathways that contribute to melanoma development and are downstream of the PI3K/PDK axis, including glucocorticoid-induced protein kinase (SGK) isoforms, glycogen synthase kinase 3 (GSK3), and Jun N-terminal kinase (JNK), cannot be excluded and are under investigation.

As previously discussed, AKT is also important and was shown to be acting independently of Timp1 in the metastatic melanoma 4C11+ cell line. In Figure 3C, we note that AKT is extremely important for the aggressive status of this cell line, since transient AKT reduction has led to a decreased *anoikis* resistance. However, the decrease of AKT in cell lines silenced for Timp1 led to a more effective reduction of *anoikis* resistance, showing that Timp1 and AKT have synergistic effects to promote cell survival (Figure 6). Taken together, this finding indicates that simultaneous inhibition of Timp1 and AKT might be a potential strategy in the fight against metastatic melanoma.

## 4. Materials and Methods

Cell culture: Non-tumorigenic melan-a melanocyte lineage was cultured at 37 °C in humidified 95% air-5% CO_2_ in RPMI pH 6.9 supplemented with 5% fetal bovine serum (Invitrogen, Carlsbad, CA, USA), 200 nM 12-phorbol-13-myristate acetate (PMA; Calbiochem, Darmstadt, Germany), 100 U/mL penicillin, and 100 U/mL streptomycin (Invitrogen) [40]. A murine melanocyte malignant transformation model was developed in our laboratory using melan-a melanocyte lineage [8].

Briefly, cell lines were established after melan-a melanocytes were submitted to 1–4 cycles of substrate impairment adhesion (namely 1C, 2C, 3C, and 4C cell lines, respectively), giving rise to cell lines representing pre-malignant cells. Pre-malignant 4C melanocytes were subjected to a new cycle of anchorage blockade and the spheroids formed were submitted to a limiting dilution, giving rise different melanoma cells lines (e.g., non-metastatic 4C11− and metastatic 4C11+ cell lines) [8]. Pre-malignant 4C melanocyte lineage and their clones (non-target, shTimp1#2, shTimp1#3), non-metastatic 4C11− and their clones (non-target, shTimp1#2, shTimp1#3), and metastatic 4C11+ melanoma cell lines and their clones (non-target, shTimp1#2, shTimp1#3) were cultured as melan-a cells, but in the absence of PMA.

Reagents: All reagents were diluted in DMSO at the following concentrations: 1 µM LY294002 (PI3K inhibitor), 1 µM GSK2334470 (PDK1 inhibitor) and 2 µM Bisindolylmaleimide I (PKC inhibitor). All reagents were purchased from Sigma-Aldrich (Sigma-Aldrich Inc., St. Louis, MO, USA).

Stable silencing of Timp1 by shRNA: Timp1 was silenced by shRNA—MISSION^®^ shRNA Plasmid DNA (pLKO.1-puro) from Sigma-Aldrich. Two plasmids carrying different shRNA sequences for Timp1 (shTimp1#2, clone ID: NM_011593.1-167s1c1, and shTimp1#3, clone ID:NM_011593.1-470s1c1) and a control plasmid containing a non-target sequence (non-mammalian shRNA control, TCR1, SHC002). These sequences were previously validated for the target. Lentiviral particles were produced by the calcium phosphate precipitation method in 293T cells, co-transfecting the lentiviral plasmids containing the shRNA sequences, with packaging plasmids. Viral supernatants were aliquoted and stored at −80 °C. Lentiviral titers were determined by limiting dilution and puromycin selection in HT1080 cells (ATCC, Manassas, VA, USA) and quantified as the number of transducing units (TU) per milliliter. These lentiviral particles were used to establish cell lines with knockdown of Timp1 or expressing the non-target shRNA sequence as a control. All transductions were performed using a MOI (multiplicity of infection) of 0.5, in the presence of 8 µg/mL polybrene. After transduction puromycin-resistant cells were selected. The target silencing was verified by RT-qPCR.

Transient silencing of AKT: Metastatic melanoma cell line 4C11+ was plated in six-well plates (5 × 10^4^ cells per well) and maintained in culture for 24 h. Nine microliters of Lipofectamine^®^ RNAiMAX Reagent (Life Technologies, Carlsbad, CA, USA) were diluted in 150 μL of Optim-MEM^®^ medium (Life Technologies). Thirty picomoles of siRNA (Ambion^TM^, Burlington, Ontario, Canada) were diluted in 150 μL of Optim-MEM^®^ medium (Life Technologies). Both solutions containing Lipofectamine^®^ RNAiMAX Reagent (Life Technologies) and siRNA (Silencer^®^ Select AKT1 (Ambion^TM^, cat. #4390771) were mixed and incubated for 5 min at room temperature. After 5 min, the solution containing the siRNA was dripped into wells. The cell lines were maintained in culture for 48 h and, after that time, cells were used for *anoikis* resistance assay. For the control, cells lines were transfected with a non-targeting negative control siRNA (Silencer^®^ Select Negative Control No. 1 siRNA, Ambion^TM^, cat. #4390843) as described above. The negative control has the same chemical modifications as Silencer^®^ Select siRNA but without targeting any RNA.

Western blotting: Subconfluent cell cultures were trypsinized, washed with PBS and membrane-enriched protein extracts were prepared using cold NP-40 lysis buffer (10% NP-40 in 100 mMNaCl and 50 mMTris pH 7.4, containing 30 mM sodium pyrophosphate, 50 mMNaF, 1 mM NaVO_4_, 2 µg/mL aprotinin, 2 µg/mL leupeptin, 2 µg/mL pepstatin, and 1 mM PMSF), kept for 15 min on ice, followed by centrifuging at 10,000 rpm for 15 min at 4 °C. The supernatant was collected and the protein concentration was measured by Bio-Rad protein assay dye reagent concentrate (Bio-Rad, Hercules, CA, USA). Equivalent amounts of protein (50 μg) were denaturated in SDS-sample buffer (240 mMTris-HCl pH 6.8, 0.8% SDS, 200 mM**β**-mercaptoethanol, 40% glycerol, and 0.02% bromophenol blue) for 5 min, then separated by electrophoresis in SDS-polyacrylamide gels and transferred to a polyvinylidenedifluoride membrane (Amersham, Piscataway, NJ, USA). After protein transfer, the membranes were blocked with 5% non-fat dry milk in PBS (10 mM phosphate buffer pH 7.2, 150 mMNaCl), incubated with the indicated antibodies: anti-GAPDH (#ABS16, Chemicon, Darmstadt, Germany), anti-actin (A2103, Sigma-Aldrich), anti-phospho-AKT^Thr308^ (#9275, Cell Signaling Technology) and anti-AKT (#4691, Cell Signaling Technology), anti-phospho-PDK1^Ser241^ (#3061, Cell Signaling Technology), anti-PDK1 (#3062, Cell Signaling Technology), anti-phospho-PKC(pan)(βIISer660) (#9371, Cell Signaling Technology), anti-PKC (sc-17769, Santa Cruz Biotechnology, Dallas, TX, USA), caspase-3 (CSB-PA05689A0Rb, Flarebio Biotech LLC, College Park, MD, USA), and Timp1 (CSB-PA024013YA01HU, Flarebio Biotech LLC) overnight at 4 °C and the signal was detected using horseradish peroxidase-conjugated anti-immunoglobulin G antibody (KPL, Gaithersburg, MD, USA) followed by development using chemiluminescence substrate (SuperSignal West Pico Chemiluminescent Substrate; Pierce Chemical, Rockford, IL, USA).

MTT assay: To estimate cell proliferation, 2 × 10^3^ cells were plated in 96-well plates, and cell viability was determined by MTT assay every 24 h. To this, MTT (5 µg/mL final concentration) (Calbiochem) was added to the culture. The cells were kept in an incubator (37 °C and 5% CO_2_) for one hour. MTT was removed from the wells by inverting the plate and 100 µL of isopropanol were added to each well (Merck, Darmstadt, Germany). After 15 min, the absorbance was measured with a spectrophotometer (OD = 620 nm) (Multiskan EX/Thermo Electron Corp., Waltham, MA, USA). The same procedure described above was carried out to determine viable cells both after treatment with the drug and *anoikis* resistance assay.

*Anoikis* resistance assay: Cell lines 4C wild-type, 4C non-target (TRC2), 4C shTimp1#2, 4C shTimp1#3, 4C11− wild-type, 4C11− non-target (TRC2), 4C11− shTimp1#2, 4C11− shTimp1#3, 4C11+ wild-type, 4C11+ non-target (TRC2), 4C11+ shTimp1#2, 4C11+ shTimp1#3, 4C11− non-target (AKTi CTL), 4C11− AKTi, 4C11+ non-target (AKTi CTL), and 4C11+ AKTi (5 × 104) were maintained for 96 h in 100 mm^2^ dishes coated with 1% agarose, with or without treatment with specific inhibitors. After 96 h, cell viability was assessed by MTT and/or analyzed for caspase-3 cleavage by Western blotting.

Colony formation assay (clonogenicity). Cell lines (10^6^) were treated or not with the inhibitors. After 24 h of treatment, cells were trypsinized and 200 cells were plated to visualize the formation of colonies. After 10 days, the colonies were washed with PBS, fixed with 3.7% paraformaldehyde for 15 min, stained with 1% toluidine blue for 5 min and washed with water. For quantification of viable cells, the cells were lysed and the dye solubilized in 1% SDS for 4 h and the absorbance at 620 nm was measured using a plate reader.

Determination of IC50 dose: To determine the IC50 dose of the drug dacarbazine, melanoma cell lines (2.5 × 10^3^) were plated in 96-well plates. Twenty-four hours after cell adhesion, serial dilution of the drug was accomplished in the following concentrations: 0, 200, 400, 600, 800, and 1000 µM in order to determine the IC50. After 48 h, cell viability was assessed by MTT assay.

Tumor growth assay: Cells (2 × 10^5^) were inoculated in the subcutaneous tissue of the dorsal region of female C57BL/6 mice (five animals per group). After 12 days, tumor formation was observed and measured every two days. On day 18, mice were sacrificed and the tumor weight determined. All procedures involving animals were performed after approval from the Research Ethics Committee of the Universidade Federal de São Paulo, Brazil (Approval number: CEP 2116/11).

Lung colonization assay: Cells (2.5 × 10^5^) were inoculated on the lateral tail vein of female C57BL/6 mice. After 21 days, the animals were sacrificed and the presence of colonies in the lung was analyzed.

Statistical analyses: All tests were conducted in biological triplicate. The results were organized into a database using the statistical program GraphPad Prism 5 (GraphPad Software Inc., La Jolla, CA, USA). The level of significance utilized was *p* < 0.05. The statistical tests performed were: Student’s *t*-test for unpaired samples, and one-way ANOVA followed by Tukey’s multiple comparison tests.

## 5. Conclusions

Our data provides evidence that Timp1 confers cell survival by activating PDK1 signaling pathway and that Timp1 and AKT have synergistic effects to confer *anoikis* resistance in metastatic melanoma cells.
